# Identification of the Prognosis Value and Potential Mechanism of Immune Checkpoints in Renal Clear Cell Carcinoma Microenvironment

**DOI:** 10.3389/fonc.2021.720125

**Published:** 2021-07-14

**Authors:** Guodong Liao, Ping Wang, Yuyong Wang

**Affiliations:** ^1^ Department of Urology, The First Affiliated Hospital, College of Medicine, Zhejiang University, Hangzhou, China; ^2^ Department of Urology, Affiliated Hangzhou First People’s Hospital, Zhejiang University School of Medicine, Hangzhou, China

**Keywords:** KIRC, CTLA4, miR-20b-5p, immune infiltration, prognosis biomarker

## Abstract

**Background:**

Kidney Renal Clear Cell Carcinoma (KIRC) is one of the most prevalent types of cancer worldwide. KIRC has a poor prognosis and, to date, immunotherapy based on immune checkpoints is the most promising treatment. However, the role of immune checkpoints in KIRC remains ambiguous.

**Methods:**

Bioinformatics analyses and qRT-PCR were performed to explore and further confirm the prognostic value of immune checkpoint genes and their correlation with immune infiltration in KIRC samples.

**Results:**

The expression of the immune checkpoint genes CD274, PDCD1LG2, HAVCR2, CTLA4, TIGFT, LAG3, and PDCD1 was upregulated in KIRC tissues. These genes were involved in the activation of the apoptosis pathway in KIRC. Low expression of CD274 and HAVCR2 and high expression of CTLA4 were associated with poor overall survival (OS), progression-free survival (PFS), and disease-free survival (DFS) of KIRC patients. The univariate and multivariate analyses revealed that CTLA4, HAVCR2, age, pTNM stage, and tumor grade were independent factors affecting the prognosis of KIRC patients. A predictive nomogram demonstrated that the calibration plots for the 3‐year and 5‐year OS probabilities showed good agreement compared to the actual OS of KIRC patients. The expression of CTLA4 and HAVCR2 were positively associated with immune cell infiltration, immune biomarkers, chemokines, and chemokine receptors. Moreover, miR-20b-5p was identified as a potential miRNA target of CTLA4 in KIRC.

**Conclusion:**

Our study clarified the prognostic value of several immune checkpoint regulators in KIRC, revealing a CTLA4/miR-20b-5p axis in the control of immune cell infiltration in the tumor microenvironment.

## Introduction

Renal cell carcinoma (RCC) is one of the most prevalent malignant tumors worldwide, accounting for 2.4% of all cancers (1). In the world, over 403,000 people are initially diagnosed with RCC every year, and 175,000 patients will die of this disease (2). Clear cell renal cell carcinoma (KIRC or ccRCC) is the most frequent histological subtype of RCC, and it accounts for most cancer-related deaths (3, 4). Due to the lack of significant clinical symptomatology, KIRC can remain clinically occult, and therefore patients are initially diagnosed in an advanced TNM stage. Late diagnosis generally correlates with lower survival, leading to a poor 5-years survival rate in KIRC patients (5). Moreover, the mortality rates of KIRC have risen to high levels and stabilized in the past ten years (6). The 5-year disease specific survival of RCC patients in stage I was about 80-95%, while it dropped sharply to less than 10% in KIRC patients in stage IV, whose median overall survival was only 10-15 months (4).

Latest studies indicated that immunotherapy based on immune checkpoint regulators is the most promising treatment for KIRC, especially in advanced stages (7). Thus, it is vital to clarify the relation between KIRC and immune infiltration, as well as to identify immune-associated mechanisms and markers for the prognosis and therapy of KIRC. Immune checkpoint molecules secreted from immune cells will inhibit the function of immune cells so that the body cannot produce an effective anti-tumor immune response, leading to immune escape and tumor formation (8). Previous studies have identified many immune checkpoints, including but not limited to SIGLEC15, CD274 (PD-L1), HAVCR2 (TIM-3), PDCD1, CTLA4, LAG3, PDCD1LG2, and TIGIT (8, 9). The PD-1 (CD279) inhibitor nivolumab improved overall survival in advanced renal cell carcinoma following prior anti-angiogenic therapy, suggesting immunotherapy as a promising strategy for the management of KIRC (10). Another meta-analysis revealed that PD-1/PD-L1 agents showed a better performance in the treatment for sarcomatoid renal cell carcinoma than sunitinib (11). However, immune checkpoint inhibitors can cause various immune-related adverse events, including adrenal insufficiency and autoimmune hepatitis (12). Thus, further studies should investigate the relationship between these immune checkpoints and their role in the prognosis and management of KIRC.

With the continuous development of gene sequencing technologies and the establishment and improvement of various tumor databases, bioinformatic research has been suggested as one of the most reliable ways to accelerate clinical and translational cancer research. Our study aimed to clarify the correlation between immune checkpoint expression, immune infiltration, and KIRC prognosis. Moreover, our results might provide additional data about the molecular mechanism of immune checkpoint regulators in immune infiltration.

## Materials and Methods

### Datasets

In order to explore the clinical significance of immune checkpoints in KIRC, we first retrieved the KIRC gene expression profile from the Cancer Genome Atlas (TCGA) database (https://portal.gdc.cancer.gov/) and Oncomine (https://www.oncomine.org/). In TCGA, the KIRC dataset (TCGA-KIRC) was downloaded for analysis, and the corresponding clinical information, including gender, tumor grade, and survival status of the patients, were also downloaded and sorted. The Oncomine datasets GSE14994, GSE6344, and GSE11151, were also downloaded to analyze the expression of immune checkpoints.

### Gene Expression Analysis

The gene expression of eight immune checkpoints was analyzed using Oncomine and TCGA. In TCGA, the relevant transcripts and expression values of the genes were extracted and visualized using the R software packages “ggplot2” and “pheatmap” (R foundation for statistical computing, 2020; version 4.0.3). In Oncomine, Student’s t-test was used to evaluate the significance and compare the gene expression of immune checkpoints between normal tissues and KIRC tissues. A p-value threshold of 0.05 and a fold-change of 2 were set to define statistically significant changes.

### Genetic Mutation, Drug Sensitivity, and Cancer-Related Pathway Analysis

The genetic mutation data were downloaded from the TCGA dataset, and the genetic mutation of eight immune checkpoints was analyzed and visualized using the “maftools” R package. We collected 265 small molecules from Genomics of Drug Sensitivity in Cancer (GDSC) to analyze the correlation between immune checkpoints and drug sensitivity. Pearson correlation analysis was used to calculate the correlation coefficient, and a p-value < 0.05 was considered statistically significant. The method of cancer-related pathway analysis has been applied as in Ye et al. (13). Immune checkpoint expression was divided into two groups, High and Low, with median expression. The significant difference of pathway activity score (PAS) between groups is evaluated by the Student’s t-test. We considered that an immune checkpoint might have an activating effect on a pathway when PAS^H^ (immune checkpoint group High) > PAS^L^ (immune checkpoint group Low); otherwise, the checkpoint might have an inhibitory effect on the pathway.

### Enrichment Analysis and Protein-Protein Interaction (PPI) Networks of Immune Checkpoints

The functional annotation of immune checkpoints was analyzed with Metascape (https://metascape.org/), a powerful tool to comprehensively analyze and interpret OMICs-based data (14). We also constructed a PPI network of immune checkpoints using GeneMANIA (https://genemania.org/), a prediction server for gene prioritization and predicting gene function (15).

### Prognosis Analysis

The Kaplan-Meier method was applied to analyze the prognosis of immune checkpoints in KIRC. p-value and hazard ratio (HR) with 95% confidence interval (CI) were calculated using a log-rank test. A predictive nomogram was constructed based on proper terms identified by univariate and multivariate cox regression analysis. A forest was used to show the p-value and HR as well as 95% CI of each variable using the “forestplot” R package.

### Clinical Tissues and qRT-PCR

We obtained 30 KIRC tissues and normal kidney tissues from patients who underwent tumor resection in the Affiliated Hangzhou First People’s Hospital. Histological diagnosis and tumor grade were assessed by three experienced pathologists following the 2010 American Joint Committee on Cancer (AJCC) staging system. All procedures were approved by the Ethics Committee of Affiliated Hangzhou First People’s Hospital, and informed consent was obtained from each patient.

Total RNA of clinical tissues was extracted using TRIzol reagent (Invitrogen; Thermo Fisher Scientific, Inc), and PrimeScript RT-polymerase (Vazyme) was used to synthesize the cDNA according to the manufacturer´s instructions. RT-qPCR was performed with SYBR-Green Premix (Qiagen GmbH) with specific PCR primers (Sangon). Glyceraldehyde-3-phosphate dehydrogenase (GAPDH) was used as an internal control. The primers of GAPDH and immune checkpoints were shown in **Supplementary Table 1**. The fold-change was calculated as previously described with the 2^−ΔΔCt^ method. The Student’s t-test was conducted to compare the expression of immune checkpoints in KIRC and normal tissues. Kaplan-Meier analysis was performed to evaluate the prognosis value of immune checkpoints in KIRC.

### Immune Infiltration Analysis

Immune infiltration of immune checkpoints in KIRC was analyzed using TIMER (https://cistrome.shinyapps.io/timer/), a web server designed for comprehensive analysis of tumor-infiltrating immune cells (16). Spearman’s correlation analysis was performed to explore the relationship between immune checkpoints and immune cell infiltration and the expression of immune biomarkers, chemokines, and chemokine receptors. A p-value of less than 0.05 was considered statistically significant.

### Construction of miRNA-mRNA Regulatory Axis

miRNAs binding to immune checkpoint genes were identified using starBase v3.0. The expression and prognosis value of miRNA in KIRC were explored as described above.

## Results

### The Expression of Immune Checkpoints in KIRC

**Figure 1:**
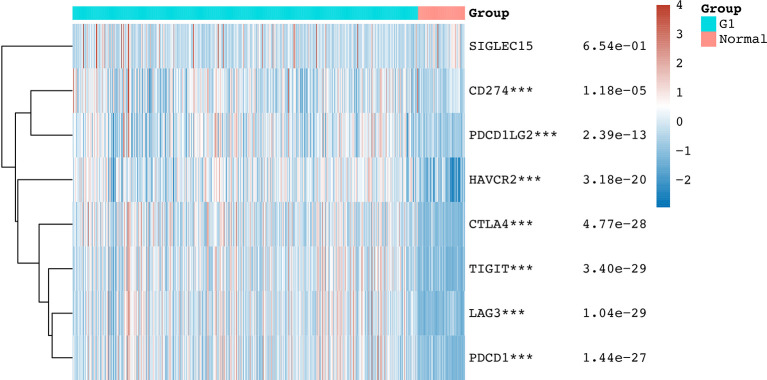
The mRNA level of immune checkpoints in KIRC. The graph shows the mRNA level of immune checkpoints in KIRC tissues compared with normal tissues. ***p < 0.001.

We initially detected the expression of immune checkpoint molecules in KIRC. According to the data from TCGA, the mRNA expression of several immune checkpoint molecules revealed that the expression of CD274 (p = 1.18e-05), CTLA4 (p = 4.77e-28), HAVCR2 (p = 3.18e-20), LAG3 (p =1.04e-29), PDCD1LG2 (p = 2.39e-13), PDCD1 (p = 1.44e-27), and TIGFT (p = 3.4e-29) were upregulated in KIRC tissues compared with normal tissues (**Figure 1**). We also detected upregulation of HAVCR2, CTLA4, and TIGIT in KIRC samples using the Oncomine dataset. The expression of HAVCR2, CTLA4, and TIGIT was upregulated in KIRC tissues compared with normal tissues with a fold change of 3.536, 11.413, and 7.749, respectively (17) (**Supplementary Table 2** and **Supplementary Figure 1**, p < 0.05). These data demonstrated extensive alteration of the expression of immune checkpoint molecules in KIRC.

### Cancer Hallmarks Analysis of Immune Checkpoints in KIRC

**Figure 2:**
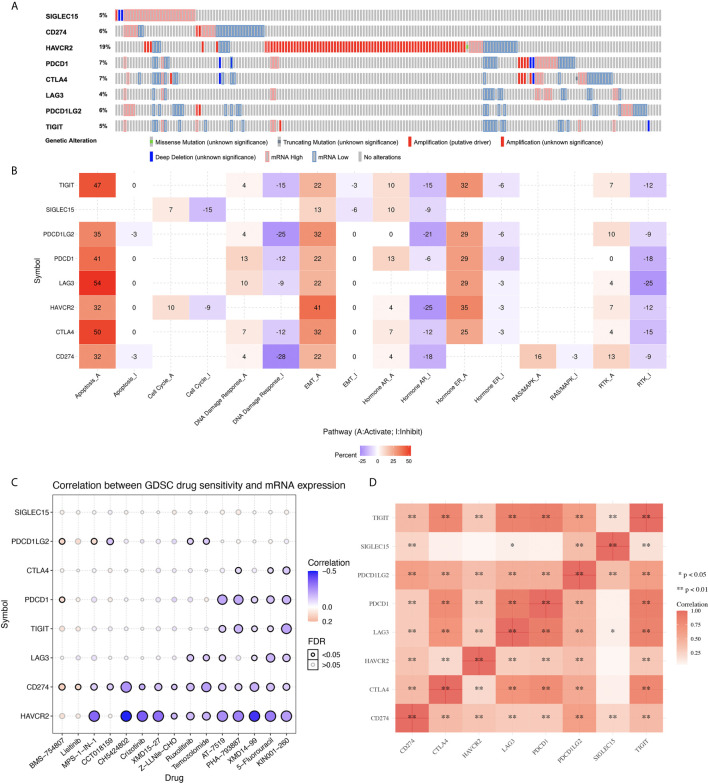
Genetic mutation landscape and drug sensitivity analysis of immune checkpoints in KIRC. **(A)** Oncoplot displaying genetic mutation landscape of immune checkpoints in TCGA KIRC cohort. **(B)** The activation and inhibition of immune checkpoints in KIRC-related pathways. **(C)** The correlation between immune checkpoints and drug or small molecules. The positive correlation means that the gene high expression is resistant to the drug, vise verse. **(D)** A heat map of the correlation between each member of immune checkpoints.

In order to investigate the role of immune checkpoint molecules in KIRC, we also performed cancer hallmarks analysis. The genetic alteration of the immune checkpoints in the TCGA-KIRC patients comprised missense mutations, truncating mutations, amplifications, deep deletions, and mRNA upregulation and downregulation (**Figure 2A**). HAVCR2 was the most commonly altered gene among all the studied immune checkpoint genes, and about 19% of the total TCGA-KIRC cases counted with a HAVCR2 genetic mutation (**Figure 2A**). Activation and inhibition of cancer hallmark pathways play a vital role in tumorigenesis and progression. Therefore, we then explored the effect of these immune checkpoint regulators in several cancer hallmark pathways in KIRC. These pathways included TSC/mTOR, RTK, RAS/MAPK, PI3K/AKT, Hormone ER, Hormone AR, EMT, DNA Damage Response, Cell Cycle, and Apoptosis pathways. The results indicated that immune checkpoints were involved in the activation of the apoptosis pathway, EMT pathway, and the inhibition of DNA damage response pathway in KIRC (**Figure 2B**). In order to identify potential therapeutic targets, a critical step is to evaluate the relation between immune checkpoints and existing drug targets. Interestingly, a drug sensitivity analysis revealed that most of these immune checkpoints are sensitive to most of the small molecules or drugs from GDSC (negative correlation, coefficient from -0.50 to -0.10) (**Figure 2C**, p<0.05). Moreover, co-expression analysis suggested a moderate to high correlation (coefficient from 0.25 to 0.80) among several immune checkpoint molecules (**Figure 2D**, p<0.05).

### Enrichment Analysis of Immune Checkpoints in KIRC

In order to clarify the immune checkpoint-associated functions in KIRC, we performed a gene enrichment analysis. As shown in **Supplementary Figure 2B**, these immune checkpoints were mainly associated with biological adhesion, immune system process, regulation of the biological process, cell proliferation, and cellular process in Gene Ontology (GO) and Kyoto Encyclopedia of Genes and Genomes (KEGG) analyses (p<0.05). Moreover, the PPI network based on immune checkpoints suggested that these immune checkpoints were mainly associated with T cell costimulation, lymphocyte costimulation, regulation of T cell, and lymphocyte activation (**Supplementary Figure 2B**, p<0.05).

### Prognosis Value of Immune Checkpoints in KIRC

**Figure 3:**
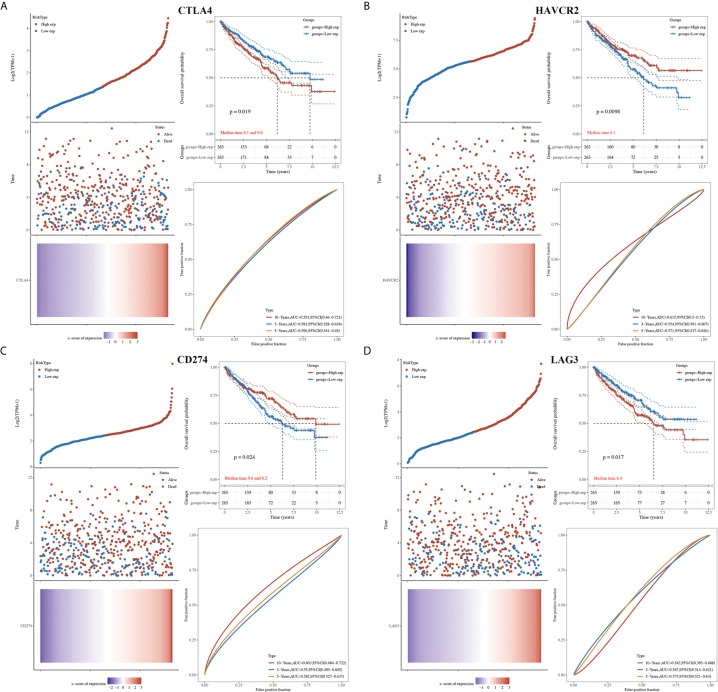
The overall survival analysis of immune checkpoints in KIRC. **(A)** The overall survival curve of CTLA4 in KIRC patients with high and low CTLA4 expression, and the risk score, survival status and gene expression of each patients, as well as time-dependent ROC. **(B)** The overall survival curve of HAVCR2 in KIRC patients with high and low HAVCR2 expression, and the risk score, survival status and gene expression of each patients, as well as time-dependent ROC. **(C)** The overall survival curve of CD274 in KIRC patients with high and low CD274 expression, and the risk score, survival status and gene expression of each patients, as well as time-dependent ROC. **(D)** The overall survival curve of LAG3 in KIRC patients with high and low LAG3 expression, and the risk score, survival status and gene expression of each patients, as well as time-dependent ROC.

Next, we explored the prognosis value of immune checkpoints in KIRC. The results suggested that CTLA4, HAVCR2 and CD274 were significantly associated with the overall survival (OS), progression free survival (PFS), and disease-free survival (DFS) of KIRC patients (**Table 1**). In OS analysis, KIRC patients with high CTLA4 expression [p = 0.019, HR (95%CI) = 1.43 (1.06-1.93)], low HAVCR2 expression [p = 0.0098, HR (95%CI) = 0.67 (0.50-0.91)], low CD274 expression [p = 0.024, HR (95%CI) = 0.71 (0.52-0.96)], high LAG3 expression [p = 0.018, HR (95%CI) = 1.44 (1.07-1.95)] had a poor OS with a 5-year AUC of 0.596, 0.571, 0.582 and 0.575, respectively (**Table 1** and **Figure 3**). In PFS analysis, KIRC patients with high CTLA4 expression [p = 0.012, HR (95%CI) = 1.68 (1.22-2.32)], low HAVCR2 expression [p = 0.0046, HR (95%CI) = 0.63 (0.46-087)], and low CD274 expression [p = 0.024, HR (95%CI) = 0.70 (0.51-0.96)] had a poor PFS with a 5-year AUC of 0.588, 0.606, and 0.576, respectively (**Table 1** and **Supplementary Figure 3**). In DFS analysis, KIRC patients with high CTLA4 expression [p = 0.00098, HR (95%CI) = 1.84 (1.25-2.72)], low HAVCR2 expression [p = 0.0046, HR (95%CI) = 0.58 (0.39-0.85)], low CD274 expression [p = 0.0092, HR (95%CI) = 0.60 (0.41-0.89)], high LAG3 expression [p = 0.014, HR (95%CI) = 1.64 (1.11-2.43)] had a poor DFS with a 5-year AUC of 0.627, 0.588, 0.592 and 0.6, respectively (**Table 1** and **Supplementary Figure 4**). These data demonstrated that CD274, HAVCR2 and CTLA4 might serve as prognostic biomarkers in KIRC.

**Table 1:** Prognosis analysis of immune checkpoints in KIRC.

Genes	Overall Survival		Progression Free Survival		Disease Free Survival	
	p-value	HR (95%CI)	p-value	HR (95%CI)	p-value	HR (95%CI)
SIGLEC15	0.928	1.01 (0.75-1.37)	0.375	0.87 (0.63-1.19)	0.862	0.97 (0.66-1.41)
**CD274**	**0.024**	**0.71 (0.52-0.96)**	**0.024**	**0.70 (0.51-0.96)**	**0.0092**	**0.60 (0.41-0.89)**
**HAVCR2**	**0.0098**	**0.67 (0.50-0.91)**	**0.0046**	**0.63 (0.46-0.87)**	**0.0046**	**0.58 (0.39-0.85)**
PDCD1	0.199	1.22 (0.90-1.64)	0.092	1.31 (0.96-1.79)	0.087	1.40 (0.95-2.05)
**CTLA4**	**0.019**	**1.43 (1.06-1.93)**	**0.0012**	**1.68 (1.22-2.32)**	**0.00098**	**1.84 (1.25-2.72)**
LAG3	0.017	1.44 (1.07-1.95)	0.075	1.33 (0.97-1.82)	0.014	1.64 (1.11-2.43)
PDCD1LG2	0.151	0.80 (0.59-1.08)	0.227	0.82 (0.60-1.13)	0.211	0.78 (0.54-1.15)
TIGIT	0.186	1.22 (0.91-1.65)	0.14	1.27 (0.92-1.73)	0.096	1.11 (0.83-1.52)

### Predictive Nomogram Based on Clinicopathologic Features and Immune Checkpoints

**Figure 4:**
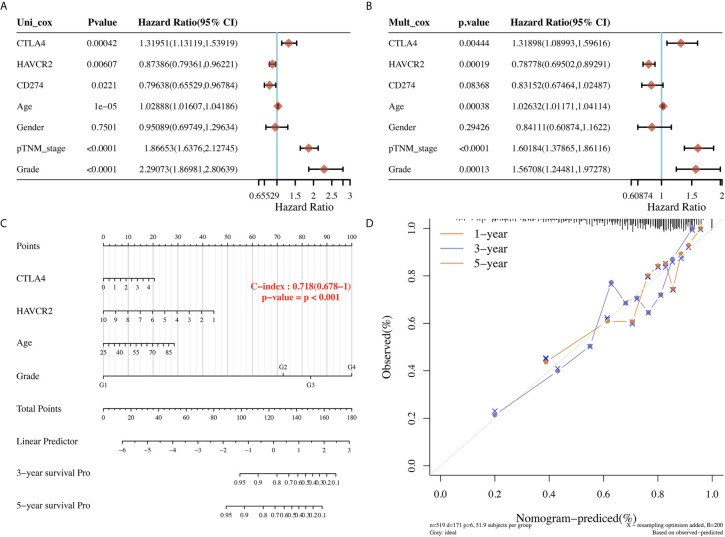
Univariate and multivariate cox regression of immune checkpoints in KIRC. **(A, B)** Univariate and multivariate cox regression of KIRC patients’ parameters and prognostic biomarkers of immune checkpoints. **(C, D)** The predictive nomogram to predict the 3-y and 5-y overall survival of KIRC cancer patients.

The univariate and multivariate analysis revealed that CTLA43 (p=0.00444), HAVCR2 (p=0.0019), age (p=0.0038), pTNM stage (p<0.0001), and tumor grade (p=0.00013) were independent factors affecting the prognosis of KIRC patients (**Figures 4A, B**). Considering clinicopathologic features and HAVCR2 and CTLA4 as potential prognostic biomarkers, we constructed a predictive nomogram to predict the 1‐year, 3‐year, and 5‐year OS rates in the discovery group using the cox regression algorithm. The nomogram demonstrated that the predicted calibration plots for 3- and 5-year OS probabilities showed good agreement compared to the actual OS of KIRC patients (**Figures 4C, D**, p<0.001).

### The Correlation Between CTLA4/HAVCR2 and Clinical Characteristics in KIRC

**Figure 5:**
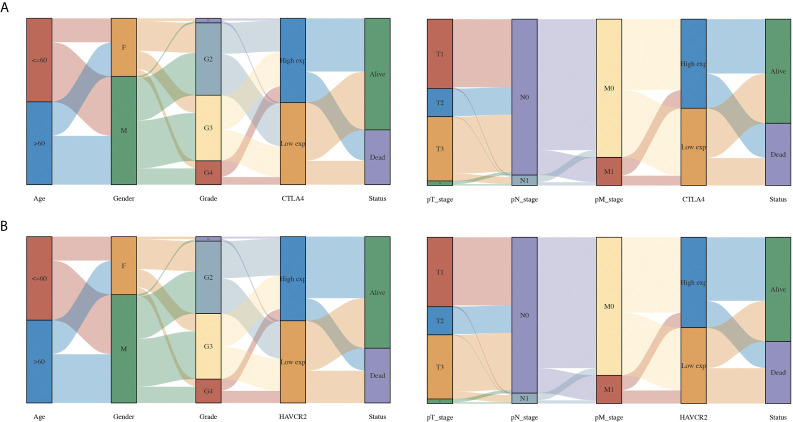
The association between immune checkpoints and the clinical parameters of KIRC patients. **(A)** The association between CTLA4 and the clinical parameters of KIRC patients. **(B)** The association between HAVCR2 and the clinical parameters of KIRC patients.

The above results revealed that CTLA4 and HAVCR2 were independent factors affecting the prognosis of KIRC and were associated with the OS, PFS, and DFS of KIRC patients. Therefore, we selected CTLA4 and HAVCR2 for further analysis. In order to explore the function of CTLA4 and HAVCR2 in KIRC, we analyzed the correlation between CTLA4 and HAVCR2 expression and the clinical characteristics of KIRC. As a result, KIRC patients with a high pT stage (p = 0.00023) had a low CTLA4 expression compared with those with a low pT stage (**Figure 5A**). Moreover, KIRC patients with a high pN stage (p = 0.04) had a lower HAVCR2 expression than those with a low pN stage (**Figure 5B**).

### Validation of the Expression and Overall Survival of CTLA4 and HAVCR2 in KIRC

**Figure 6:**
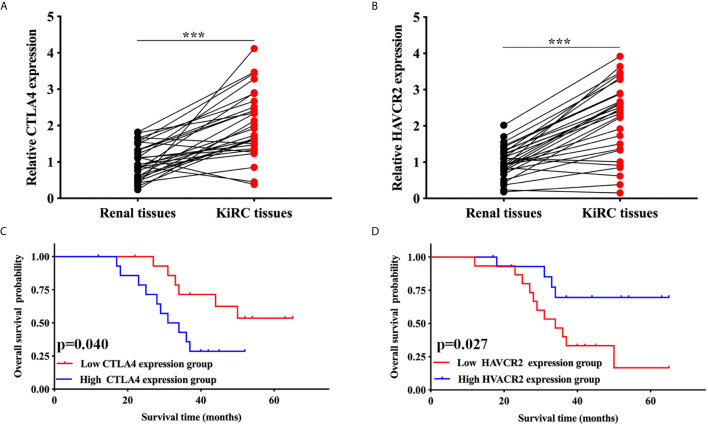
Validation of the expression and overall survival of CTLA4/HAVCR2 in KIRC. **(A, B)** The expression of CTLA4/HAVCR2 in KIRC and normal renal tissues. **(C, D)** The overall survival curve of CTLA4/HAVCR2 in KIRC patients in high and low expression cohort. ***p < 0.001.

We then performed qRT-PCR to further confirm the expression of CTLA4 and HAVCR2 in KIRC. As expected, the relative mRNA level of CTLA4 (p < 0.001, **Figure 6A**) and HAVCR2 (p < 0.001, **Figure 6B**) in KIRC samples was elevated compared to normal renal tissues. In addition, we also studied the overall survival of these patients. The data indicated a poor overall survival in those patients with high CTLA4 expression (p = 0.040, **Figure 6C**) and low HAVCR2 expression (p = 0.027, **Figure 6D**), consistently with previous results.

### The Correlation Between CTLA4/HAVCR2 and Immune Infiltration in KIRC

**Figure 7:**
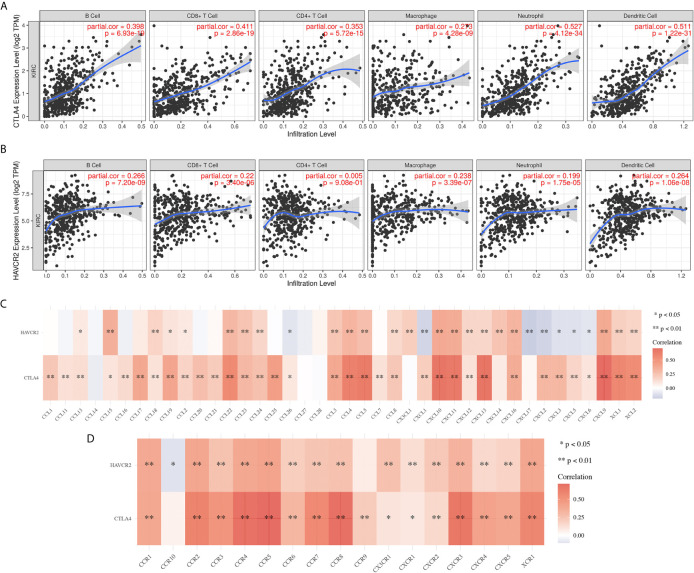
The correlation between immune checkpoints and immune infiltration in KIRC(TIMER). **(A, B)** The correlation between the expression of CTLA4 and HAVCR2 and the abundance of CD8+ T cells, CD4+ T cells, Macrophage, Neutrophils and Dendritic cells. **(C, D)** The correlation between SCNA of CTLA4 and HAVCR2 and immune cell infiltration. SCNA, somatic copy number alterations; *P < 0.05, **P < 0.01.

The immune infiltration grade is an independent predictor of sentinel lymph node status and survival in cancers (4, 18, 19). In our study, we found a significant correlation between CTLA4 and HAVCR2 and immune infiltration in KIRC samples. CTLA4 showed positive correlation with the abundance of B cells (Cor = 0.398, p = 6.93E-10), CD8+ T cells (Cor = 0.411, p = 2.86E-19), CD4+ T cells (Cor = 0.353, p = 5.72E-15), macrophages (Cor = 0.273, p = 4.28E-9), neutrophils (Cor = 0.527, p = 4.21E-34) and dendritic cells (Cor = 0.511, p = 1.22E-31) (**Figure 7A**). Similarly, HAVCR2 showed a positive correlation with the abundance of B cells, CD8+ T cells, CD4+ T cells, macrophages, neutrophils, and dendritic cells (**Figure 7B**; all p<0.05). Moreover, the expression of CTLA4 and HAVCR2 were positively correlated with most biomarkers of immune cells, including the biomarkers of CD8+ T cells, T cells (general), B cells, monocytes, TAMs, M1 macrophages, M2 macrophages, neutrophils, natural killer (NK) cells, dendritic cells (DCs), T-helper 1 (Th1) cells, T-helper 2 (Th2) cells, follicular helper T (Tfh) cells, T-helper 17 (Th17) cells, Tregs, and exhausted T cells (**Table 2**). A previous study revealed that chemokines and their receptors play a vital role in immune infiltration (20). In our study, we found that the expression of CTLA4 and HAVCR2 was positively correlated with the expression chemokines as well as chemokines receptors (**Figures 7C, D**). This evidence indicated a possible association between CTLA4/HAVCR2 and immune infiltration in KIRC patients.

**Table 2:** Correlation analysis between HAVCR2/CTLA4 and gene biomarkers of immune cells in KIRC.

Immune cells	Biomarkers	HAVCR2		CTLA4	
		Cor	P-value	Cor	P-value
CD8+ T cell	CD8A	0.367	***	0.674	***
	CD8B	0.359	***	0.621	***
T cell (general)	CD3D	0.311	***	0.726	***
	CD3E	0.333	***	0.742	***
	CD2	0.342	***	0.746	***
B cell	CD19	0.094	*	0.483	***
	CD79A	0.165	***	0.478	***
Monocyte	CD86	0.356	***	0.611	***
	CD115(CSF1R)	0.292	***	0.445	***
TAM	CCL2	0.075	0.0841	0.176	***
	CD68	0.314	***	0.286	***
	IL10	0.319	***	0.455	***
M1 Macrophage	INOS (NOS2)	0.09	*	-0.054	0.215
	IRF5	0.325	***	0.442	***
	COX2(PTGS2)	-0.1	*	0.075	0.0831
M2 Macrophage	CD163	0.315	***	0.307	***
	VSIG4	0.243	***	0.307	***
	MS4A4A	0.3	***	0.368	***
Neutrophils	CD66b (CEACAM8)	0.053	0.22	0.057	0.189
	CD11b (ITGAM)	0.312	***	0.397	***
	CCR7	0.244	***	0.558	***
Natural killer cell	KIR2DL1	0.033	0.441	0.057	0.188
	KIR2DL3	0.069	0.111	0.138	**
	KIR2DL4	0.076	0.0809	0.421	***
	KIR3DL1	0.058	0.18	0.056	0.199
	KIR3DL2	0.099	0.0224	0.187	***
	KIR3DL3	0.007	0.864	0.11	*
	KIR2DS4	-0.047	0.279	0.073	0.0937
Dendritic cell	HLA-DPB1	0.422	***	0.591	***
	HLA-DQB1	0.251	***	0.44	***
	HLA-DRA	0.445	***	0.582	***
	HLA-DPA1	0.439	***	0.569	***
	BDCA-1(CD1C)	0.214	***	0.285	***
	BDCA-4(NRP1)	0.107	*	-0.098	*
	CD11c (ITGAX)	0.299	***	0.582	***
Th1	T-bet (TBX21)	0.189	***	0.423	***
	STAT4	0.17	***	0.664	***
	STAT1	0.368	***	0.585	***
	IFN-g (IFNG)	0.291	***	0.672	***
	TNF-a (TNF)	0.12	**	0.314	***
Th2	GATA3	-0.053	0.224	0.318	***
	STAT6	0.161	***	0.096	*
	STAT5A	0.323	***	0.511	***
	IL13	-0.033	0.448	0.261	***
Tfh	BCL6	-0.112	**	0.228	***
	IL21	0.11	*	0.247	***
Th17	STAT3	0.212	***	0.123	**
	IL17A	-0.009	0.836	0.125	**
Treg	FOXP3	0.149	***	0.705	***
	CCR8	0.26	***	0.685	***
	STAT5B	0.204	***	0.007	0.875
	TGFb (TGFB1)	0.098	*	0.133	**
T cell exhaustion	PD-1 (PDCD1)	0.309	***	0.75	***
	CTLA4	0.205	***	1	***
	LAG3	0.294	***	0.707	***
	TIM-3 (HAVCR2)	1	***	0.205	***
	GZMB	0.124	**	0.445	***

*p < 0.05, **p < 0.01, ***p < 0.001.

### miRNA-mRNA Regulatory Network

**Figure 8:**
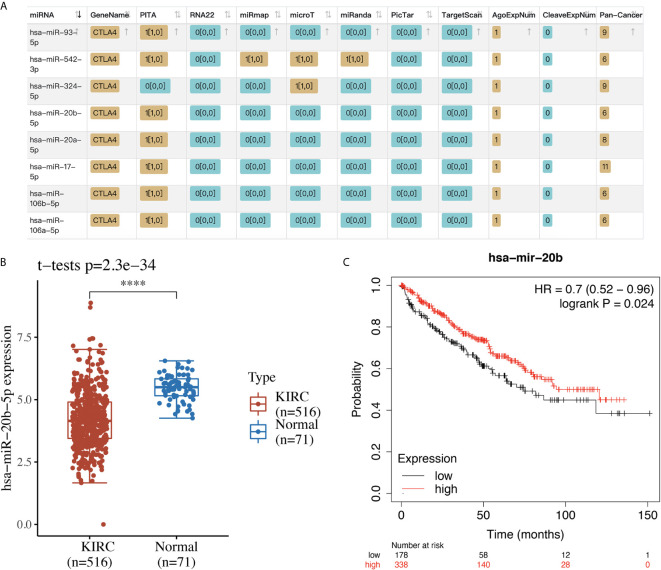
The miRNA target of CTLA4 in KIRC. **(A)** The potential miRNA targets of CTLA4 in KIRC identified by starBase. **(B)** The expression of miR-20b-5p in KIRC tissues and normal renal tissues. **(C)** The overall survival curve of KIRC patients with high and low expression of miR-20b-5p. ****p < 0.0001.

We selected CTLA4 to further analyze its potential as a therapeutic target and its molecular mechanism in KIRC. Using starBase, we searched for miRNA targets of CTLA4 in KIRC. As a result, a total of eight miRNAs (miR-93-5p, miR-542-3p, miR-324-5p, miR-20b-5p, miR-20a-5p, miR-17-5p, miR-106b-5p and miR-106a-5p) were identified as potential targets of CTLA4 in KIRC (**Figure 8A**). Next, we verified the expression and prognosis value of these eight miRNA targets, and we found that miR-20b-5p was upregulated in KIRC and associated with better prognosis (**Figures 8B, C**), suggesting miR-20b-5p as the most potentially relevant target of CTLA4 in KIRC.

## Discussion

Previous studies revealed that immune checkpoint regulators were correlated with many biological processes, thus affecting the clinical outcomes of cancer patients (21, 22). It is suggested that immune checkpoints could act as markers to predict the prognosis of many cancers, including renal cell carcinoma, lung cancer, and breast cancer (23–25). However, the prognostic value of immune checkpoints and their association with immune infiltration of KIRC remain unclear. Thus, this study aimed to detect the role of these immune checkpoints in the prognosis and immune infiltration of KIRC.

We first explored the expression level of several immune checkpoint molecules in KIRC samples. We found that the mRNA level of most of these immune checkpoints, including CD274, PDCD1LG2, HAVCR2, CTLA4, TIGFT, LAG3, and PDCD1, was altered in KIRC patients. These immune checkpoints might play a vital role in the oncogenesis and progression of KIRC. As expected, further analysis revealed that these immune checkpoints were involved in the activation of the apoptosis pathway in KIRC. Therefore, immune checkpoints may inhibit oncogenesis and progression by activating the apoptosis pathway.

Moreover, our study also found that CTLA4 and HAVCR2 acted as prognostic biomarkers in KIRC and were associated with overall survival (OS), progression-free survival (PFS), and disease-free survival (DFS) of KIRC patients. In agreement with our results, previous studies have suggested certain immune checkpoints as a prognostic biomarker of many cancers. HAVCR2 was a prognostic biomarker for gastric cancer and was negatively associated with OS (26), and two independent studies revealed that HAVCR2 was a diagnostic and prognostic biomarker of osteosarcoma (27) and large B-cell lymphoma (28).

Another important finding of our study is that a significant correlation was obtained between the expression of CTLA4 and HAVCR2 and immune cells, immune biomarkers, chemokines, and chemokine receptors. All these factors play a critical function in controlling tumor immune infiltration, anticancer immunity, and other biological processes, thus affecting the prognosis of the patients. For example, previous studies indicated that tumor-infiltrating CD8+ T cells determined poor prognosis and immune evasion (29) and B cells predicted dismal survival and worse treatment response in KIRC (30). Another bioinformatics study suggested that low mRNA levels of the chemokines CXCL1/2/3/5/13 were associated with a significantly better prognosis in KIRC (4).

## Conclusion

Our study performed a comprehensive analysis of the prognostic value of immune checkpoints in KIRC and their association with immune infiltration. Our results identified a CTLA4/miR-20b-5p axis in the control of immune infiltration in the tumor microenvironment.

## Data Availability Statement 

The original contributions presented in the study are included in the article/**Supplementary Material**. Further inquiries can be directed to the corresponding author.

## Ethics Statement

The studies involving human participants were reviewed and approved by Affiliated Hangzhou First People’s Hospital. The patients/participants provided their written informed consent to participate in this study.

## Author Contributions

GL performed data analysis work and aided in writing the manuscript. YW designed the study, assisted in writing the manuscript. PW edited the manuscript. All authors contributed to the article and approved the submitted version.

## Funding

This study was funded by National Natural Science Foundation of China (No.81772270).

## Conflict of Interest

The authors declare that the research was conducted in the absence of any commercial or financial relationships that could be construed as a potential conflict of interest.

## Supplementary Material

Supplementary Figure 1 The mRNA level of immune checkpoints in KIRC (Oncomine). The graph shows the numbers of datasets with statistically significant mRNA over-expression (red) or down-regulated expression (blue) of the target gene with a p-value of 0.05 and fold change of 2.

Supplementary Figure 2 The functional analysis of immune checkpoints in KIRC. **(A)** Heatmap of GO and KEGG enriched terms. **(B)** Protein-protein interaction network of immune checkpoints networks.

Supplementary Figure 3 The progression free survival analysis of immune checkpoints in KIRC. **(A)** The progression free survival curve of CTLA4 in KIRC patients with high and low CTLA4 expression, and the risk score, survival status and gene expression of each patients, as well as time-dependent ROC. **(B)** The progression free survival curve of HAVCR2 in KIRC patients with high and low HAVCR2 expression, and the risk score, survival status and gene expression of each patients, as well as time-dependent ROC. **(C)** The progression free curve of CD274 in KIRC patients with high and low CD274 expression, and the risk score, survival status and gene expression of each patients, as well as time-dependent ROC.

Supplementary Figure 4 The disease-free survival analysis of immune checkpoints in KIRC. **(A)** The disease-free survival curve of CTLA4 in KIRC patients with high and low CTLA4 expression, and the risk score, survival status and gene expression of each patients, as well as time-dependent ROC. **(B)** The disease-free survival curve of HAVCR2 in KIRC patients with high and low HAVCR2 expression, and the risk score, survival status and gene expression of each patients, as well as time-dependent ROC. **(C)** The disease-free survival curve of CD274 in KIRC patients with high and low CD274 expression, and the risk score, survival status and gene expression of each patients, as well as time-dependent ROC. **(D)** The disease-free survival curve of LAG3 in KIRC patients with high and low LAG3 expression, and the risk score, survival status and gene expression of each patients, as well as time-dependent ROC.

## Abbreviations

KIRC, Clear cell renal cell carcinoma; TLR3, Toll-like receptors 3; GO, Gene Ontology; KEGG, Kyoto Encyclopedia of Genes and Genomes; BP, Biological processes; CC, Cellular components; MF, Molecular functions; GSEA, Gene Set Enrichment Analysis; OS, overall survival.
