# Supplementary figures and images for: Identification of the Prognosis Value and Potential Mechanism of Immune Checkpoints in Renal Clear Cell Carcinoma Microenvironment
Source: Front Oncol. 2021 Jul 14;11:720125. doi: 10.3389/fonc.2021.720125 (PMC8317210; doi:10.3389/fonc.2021.720125)

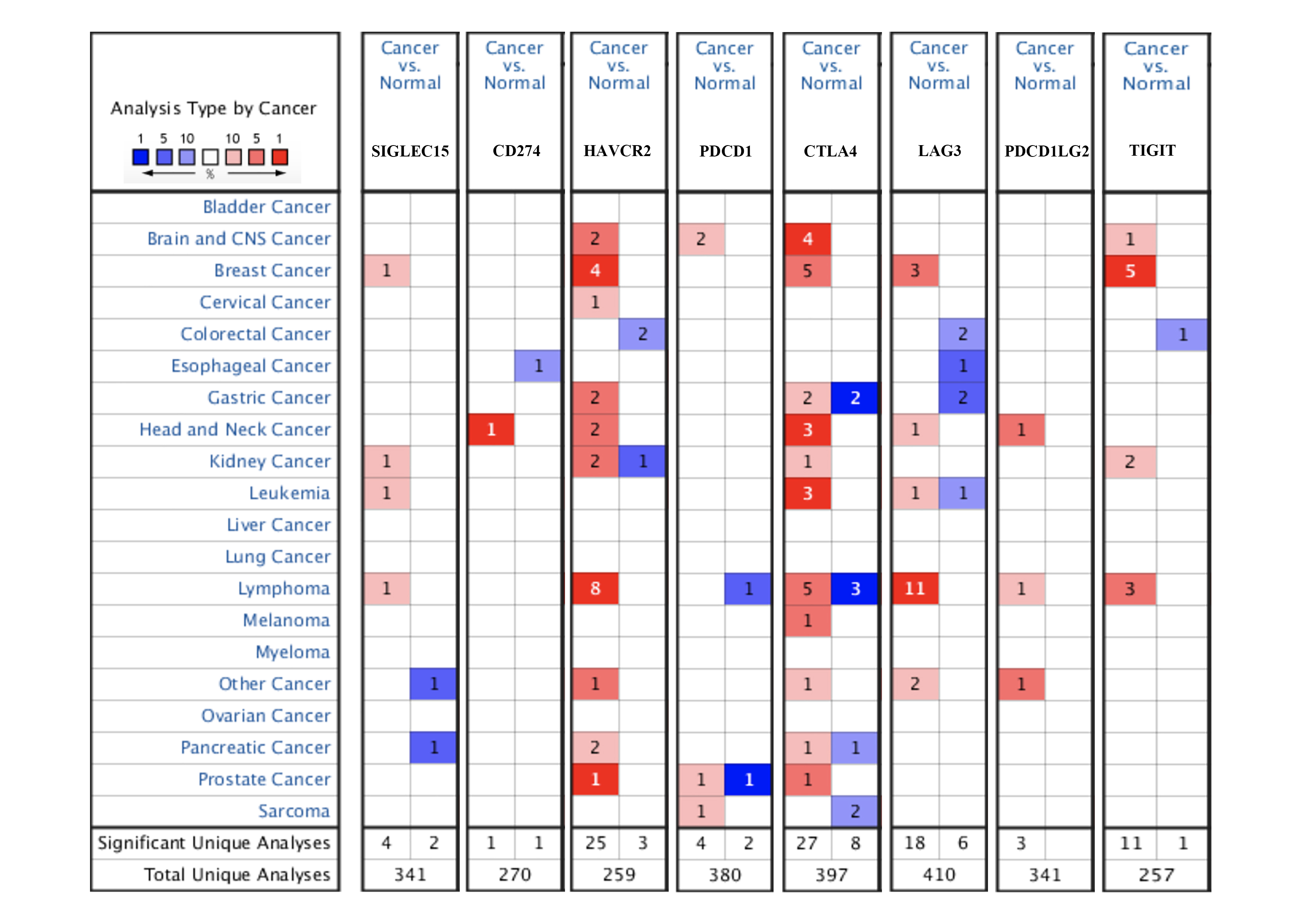

Supplement: Supplementary Figure 1 — The mRNA level of immune checkpoints in KIRC (Oncomine). The graph shows the numbers of datasets with statistically significant mRNA over-expression (red) or down-regulated expression (blue) of the target gene with a p-value of 0.05 and fold change of 2. [file Image_1.jpeg]

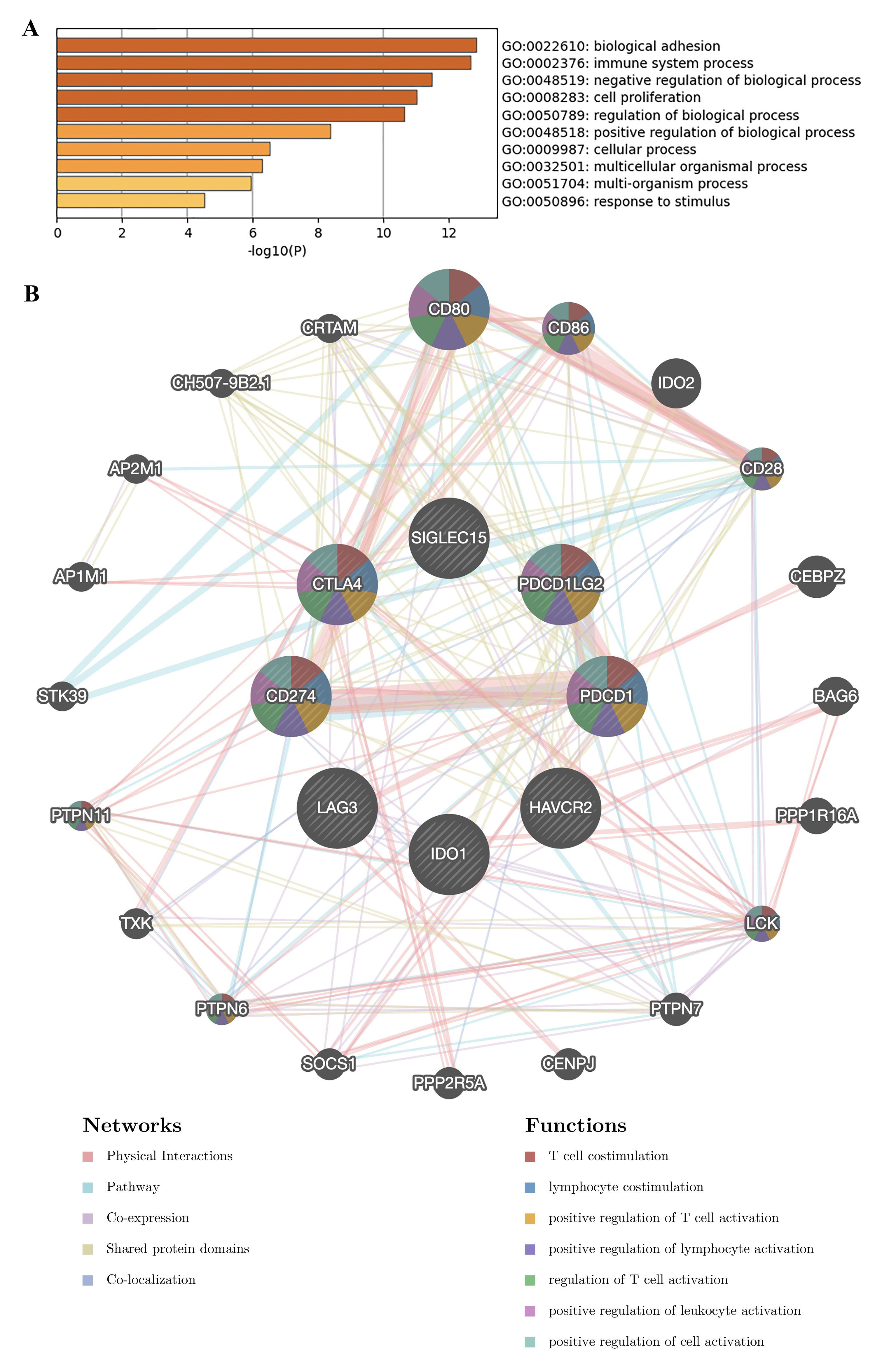

Supplement: Supplementary Figure 2 — The functional analysis of immune checkpoints in KIRC. (A) Heatmap of GO and KEGG enriched terms. (B) Protein-protein interaction network of immune checkpoints networks. [file Image_2.jpeg]

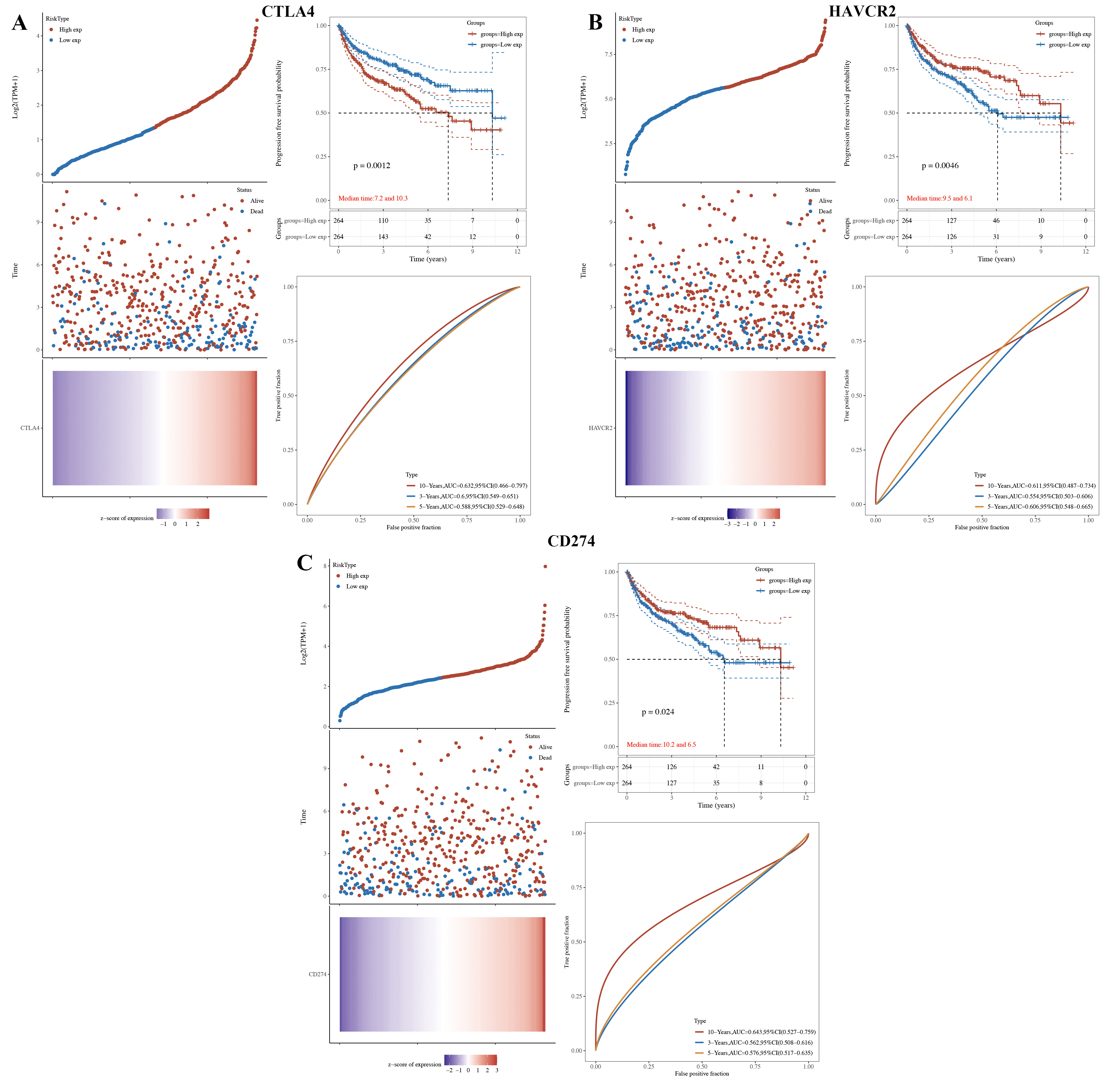

Supplement: Supplementary Figure 3 — The progression free survival analysis of immune checkpoints in KIRC. (A) The progression free survival curve of CTLA4 in KIRC patients with high and low CTLA4 expression, and the risk score, survival status and gene expression of each patients, as well as time-dependent ROC. (B) The progression free survival curve of HAVCR2 in KIRC patients with high and low HAVCR2 expression, and the risk score, survival status and gene expression of each patients, as well as time-dependent ROC. (C) The progression free curve of CD274 in KIRC patients with high and low CD274 expression, and the risk score, survival status and gene expression of each patients, as well as time-dependent ROC. [file Image_3.jpeg]

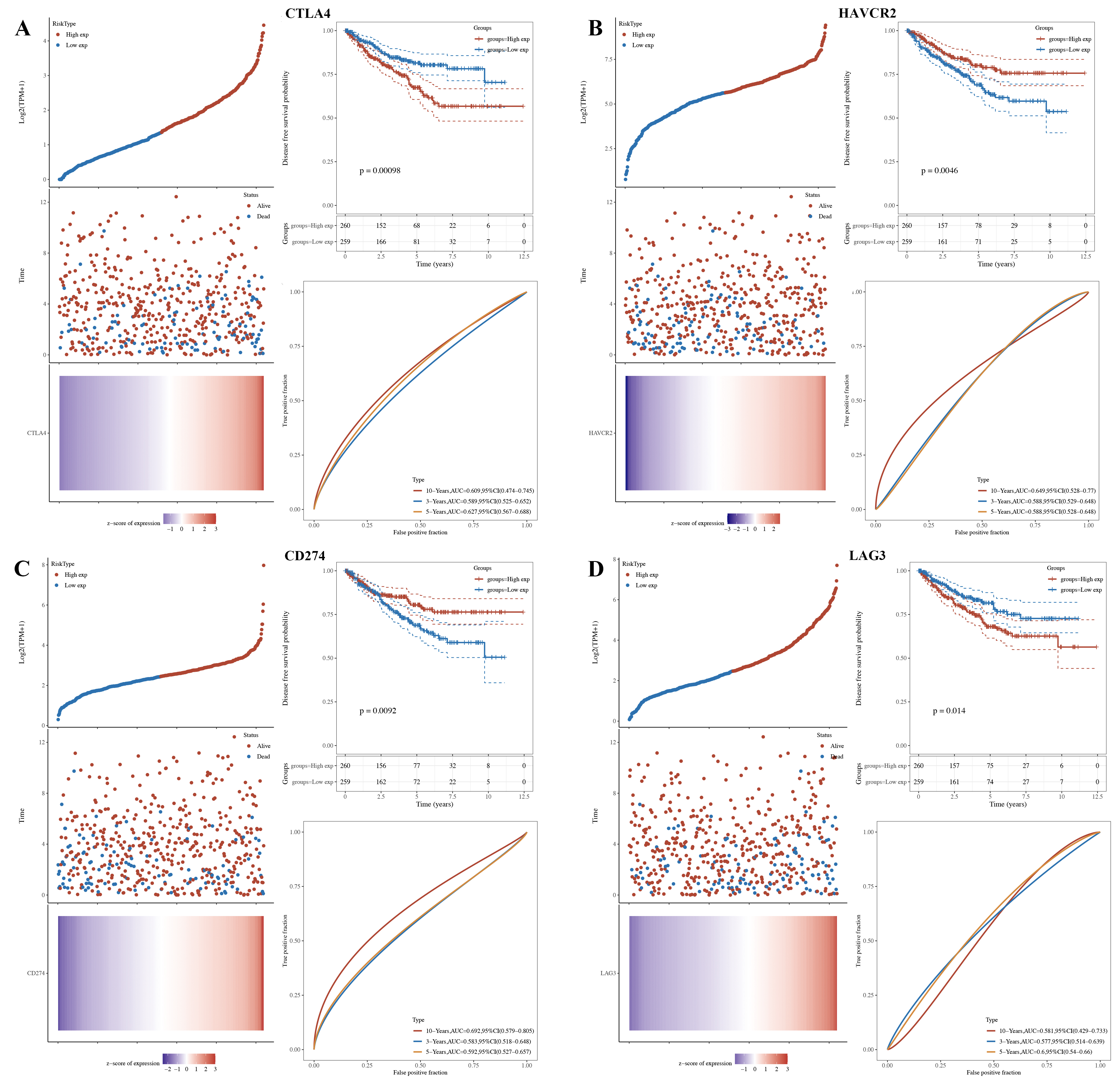

Supplement: Supplementary Figure 4 — The disease-free survival analysis of immune checkpoints in KIRC. (A) The disease-free survival curve of CTLA4 in KIRC patients with high and low CTLA4 expression, and the risk score, survival status and gene expression of each patients, as well as time-dependent ROC. (B) The disease-free survival curve of HAVCR2 in KIRC patients with high and low HAVCR2 expression, and the risk score, survival status and gene expression of each patients, as well as time-dependent ROC. (C) The disease-free survival curve of CD274 in KIRC patients with high and low CD274 expression, and the risk score, survival status and gene expression of each patients, as well as time-dependent ROC. (D) The disease-free survival curve of LAG3 in KIRC patients with high and low LAG3 expression, and the risk score, survival status and gene expression of each patients, as well as time-dependent ROC. [file Image_4.jpeg]
