# Supplementary material for: Identification of the Prognosis Value and Potential Mechanism of Immune Checkpoints in Renal Clear Cell Carcinoma Microenvironment
Source: Front Oncol. 2021 Jul 14;11:720125. doi: 10.3389/fonc.2021.720125 (PMC8317210; doi:10.3389/fonc.2021.720125)
Supplement: Supplementary file 5 [file Table_1.docx]

**Supplementary Table 1. The primers for qRT-PCR in the current study**

| **Gene** | **Primers** |
| --- | --- |
| GAPDH  CTLA4  HAVCR2 | Forward: GCACCGTCAAGGCTGAGAAC  Reverse: TGGTGAAGACGCCAGTGGA  Forward: TTTCTTCTCTTCATCCCTGTCTTCTGC  Reverse: TAAATCTGGGTTCCGTTGCCTATGC  Forward: CTGCTGCTGCTGCTGCTACTAC  Reverse: CACATTCTCTATGGTCAGGGACACATC |

**Supplementary Table 2. The mRNA levels of immune checkpoints in KIRC based on Oncomine cohort**

| TLR | | Cancer Type | Fold Change | P value | t-test | Reference |
| --- | --- | --- | --- | --- | --- | --- |
| SIGLEC15 | NA | | NA | NA | NA | NA |
| CD274 | NA | | NA | NA | NA | NA |
| HAVCR2 | Clear Cell Renal Cell Carcinoma | | 3.536 | 3.93E-4 | 4.798 | PMID:19445733 |
| PDCD1 | NA | | NA | NA | NA | NA |
| CTLA4 | Clear Cell Renal Cell Carcinoma | | 11.413 | 9.32E-5 | 6.158 | PMID:19445733 |
| LAG3 | NA | | NA | NA | NA | NA |
| PDCD1LG2 | NA | | NA | NA | NA | NA |
| TIGIT | Clear Cell Renal Cell Carcinoma | | 7.749 | 0.001 | 4.793 | PMID:19445733 |
